# Tubeimoside I Inhibits Cell Proliferation and Induces a Partly Disrupted and Cytoprotective Autophagy Through Rapidly Hyperactivation of MEK1/2-ERK1/2 Cascade via Promoting PTP1B in Melanoma

**DOI:** 10.3389/fcell.2020.607757

**Published:** 2020-12-17

**Authors:** Juan Du, Zhen Dong, Li Tan, Mengqin Tan, Fang Zhang, Kui Zhang, Guangzhao Pan, Chongyang Li, Shaomin Shi, Yanli Zhang, Yaling Liu, Hongjuan Cui

**Affiliations:** ^1^Department of Dermatology, The Third Hospital of Hebei Medical University, Shijiazhuang, China; ^2^State Key Laboratory of Silkworm Genome Biology, Institute of Sericulture and Systems Biology, College of Sericulture and Textile and Biomass, Chongqing, China; ^3^Cancer Center, Reproductive Medicine Center, Medical Research Institute, Southwest University, Chongqing, China; ^4^NHC Key Laboratory of Birth Defects and Reproductive Health (Chongqing Key Laboratory of Birth Defects and Reproductive Health, Chongqing Population and Family Planning Science and Technology Research Institute), Chongqing, China; ^5^Department of Nuclear Medicine, The Third Hospital of Hebei Medical University, Shijiazhuang, China

**Keywords:** tubeimoside-1, melanoma, MEK1/2-ERK1/2 cascade, autophagy, PTP1B

## Abstract

Tubeimoside I (TBMS1), also referred to as tubeimoside A, is a natural compound extracted from the plant Tu Bei Mu (*Bolbostemma paniculatum*), which is a traditional Chinese herb used to treat multiple diseases for more than 1,000 years. Studies in recent years reported its anti-tumor activity in several cancers. However, whether it is effective in melanoma remains unknown. In the current study, we discovered that TBMS1 treatment inhibited melanoma cell proliferation *in vitro* and tumorigenecity *in vivo*. Besides, we also observed that TBMS1 treatment induced a partly disrupted autophagy, which still remained a protective role, disruption of which by chloroquine (CQ) or 3-methyladenine (3-MA) enhanced TBMS1-induced cell proliferation inhibition. CQ combined with TBMS1 even induced cellular apoptosis. BRAF(V600E) mutation and its continuously activated downstream MEK1/2-ERK1/2 cascade are found in 50% of melanomas and are important for malanomagenesis. However, hyperactivating MEK1/2-ERK1/2 cascade can also inhibit tumor growth. Intriguingly, we observed that TBMS1 rapidly hyperactivated MEK1/2-ERK1/2, inhibition of which by its inhibitor SL-327 rescued the anti-cancerous effects of TBMS1. Besides, the targets of TBMS1 were predicted by the ZINC Database based on its structure. It is revealed that protein-tyrosine phosphatase 1B (PTP1B) might be one of the targets of TBMS1. Inhibition of PTP1B by its selective inhibitor TCS401 or shRNA rescued the anti-cancerous effects of TBMS1 in melanoma cells. These results indicated that TBMS1 might activate PTP1B, which further hyperactivates MEK1/2-ERK1/2 cascade, thereby inhibiting cell proliferation in melanoma. Our results provided the potentiality of TBMS1 as a drug candidate for melanoma therapy and confirmed that rapidly hyperactivating an oncogenic signaling pathway may also be a promising strategy for cancer treatment.

## Introduction

Melanoma derives from melanocytes, a kind of pigment-producing cells, and which mainly locate in the skin (Gigliofiorito et al., 2014). Among all types of skin cancers, melanoma is the most aggressive one and accounts for 62.1% of skin cancer death (Siegel et al., 2019). With its incidence increasing more than double over past decades, over 60,000 people died from melanoma in 2018 (Langley et al., 2018). Surgical resection is still the dominating treatment for melanoma (Molina et al., 2020). Since melanomas without nodal metastases are more common, reasonable adjuvant chemotherapy could provide benefits in preventing tumor development (Grob et al., 2018). However, for advanced melanoma patients, chemotherapy is one of the important treatments (Rozeman et al., 2018), but resistance to chemotherapy has become as one of the major problems for malignant melanoma treatment. Therefore, it is urgent to find some more effective and specific targeted therapeutic drugs for this disease.

Among a number of the therapeutic methods, traditional Chinese herbs occupy as one of the main treatments for cancers in China. The dry tuber of *Bolbostemma paniculatum*, also known as Tu Bei Mu, is a traditional Chinese herb used for more than 1,000 years and has plenty of biomedical activities including detoxication, anti-inflammatory diseases and anti-cancers (Islam et al., 2019). However, the effective components in this herb have not been identified. Recent studies showed that four triterpenoid saponins extracted from Tu Bei Mu possess biological activities: tubeimoside (TBMS) I, II, III, and V. Intriguingly, TBMSs are proved to exert the anti-tumor action in a number of cancers. Among them, TBMS I (TBMS1) is the most deeply studied one. Recently TBMS1 has been reported to be effective in many malignant tumors, such as lung cancer, non-small cell lung cancer, breast cancer, colorectal cancer, oral squamous cell carcinoma and so on (Bian et al., 2015; Hao et al., 2015; Shi et al., 2018; Wu et al., 2018; Jiang et al., 2019). Its anti-cancer effect may be realized by activating a series of biomedical effects, such as cell proliferation inhibition, cell circle arrest, apoptosis and autophagy, via various molecular pathways such as MAPK-JUK, Wnt/β-catenin, AKT or miR-126-5p pathways (Chen et al., 2012; Huang et al., 2015; Feng et al., 2018; Nakatsukasa et al., 2018). However, until now, the efficiency of TBMS1 in melanoma remains unknown.

Therefore, we wonder whether TBMS1 is a promising anti-cancer drug against melanoma. In this study, we evaluated the anti-melanoma effect of TBMS1 both *in vivo* and *in vitro*, and tried to explore the underlying molecular mechanisms, including the important signaling pathways and its probable targets.

## Materials and Methods

### Cell Culture

Human melanoma A375 cells were obtained from the American Type Culture Collection (ATCC, Manassas, VA, United States) and MV3 cells were from the University Hospital of Nijmegen (van Muijen et al., 1991). A375 cells were cultured by using the Dulbecco’s modified Eagle’s Medium (DMEM, Biological Industries, Cromwell, United States), and MV3 cells were cultured in RPMI-1640 (Biological Industries). Both kinds of mediums were supplemented with 10% fetal bovine serum (FBS, Biological Industries) and 1% penicillin and streptomycin (P/S, Invitrogen, Califonia, CA, United States). Cells were cultured at 37°C with 5% CO2 in a humidified incubator (Sanyo, Osaka, Japan).

### Drug Treatment

Tubeimoside I (TBMS1) with purity more than 99% was obtained from the Must Bio-Technology Co., Ltd. (Cat. No.:102040-03-9; Chengdu, China). SL327 was obtained from the APExBIO (Cat. No.: A1894; Boston, United States). All the above drugs were dissolved in dimethylsulfoxide (DMSO; Sigma-Aldrich, Merck, St. Louis, Missouri, United States). TCS401 was obtained from Cayman (Cat. No.: 20393; Ann Arbor, Michigan, United States) and was dissolved in 0.1 M NaOH. 3-MA was obtained from the MedChemExpress (Cat. No.: HY-19312; Shanghai, China). Chloroquine diphosphate salt was purchased from the Sigma-Aldrich (Cat. No.: C6628). These two drugs were dissolved in deionized water. Cell morphology was taken by an Olympus inverted microscopy (Olympus, Japan).

### Cell Viability Assay

Methyl thiazolyl tetrazolium (MTT, Sigma-Aldrich) assay was performed to investigate the cell viability. MV3 and A375 cells were cultured in 96-well plates overnight (800 cells in 200 μl medium per well) and then were treated with TBMS1 alone or combined with other drugs in different concentrations. DMSO was used as control. At time points (every other day), cells were incubated by adding 20 μl 5 mg/ml MTT per well in a 37°C incubator for 2 h. Then the medium was removed and 200 μl DMSO was added to each well in order to dissolve the formazan. A microplate reader (Thermo Fisher, Waltham, MA, United States) was used to measure the optical density (OD) of each well by spectrophotometry at 560 nm. Data were analyzed and IC50 was calculated by the Graphpad Prism 6.0 (San Diego, United States).

### BrdU Staining

2 × 10^4^ melanoma cells per well were cultured in 24-well plates overnight in a 37°C incubator and then were treated with TBMS1 alone or combined with other drugs for another 48 h. DMSO was used as control. After 48 h, 10 μg/ml BrdU (Sigma Aldrich) was added into cells for 2 h and then fixed with 4% paraformaldehyde for 15 min. Then cells was treated with 2 M HCl for 30 min and then followed by 0.3% Triton X-100 treatment for 15 min. Subsequently, cells were blocked with 10% goat serum in the PBST buffer (ZSGB-Bio, Beijing, China). Cells were then incubated with BrdU primary antibody (Abcam, Cambridge, MA, United States) and next with secondary antibody (Life Sciences, New York, NY, United States). Before being observed by microscopy, cells were stained with Hoechst 33258 (Life Sciences). BrdU-positive melanoma cells in random fields were counted and calculated.

### Western Blot Analysis

Cells were collected and then lysed for 40 min in a RIPA lysis buffer (Beyotime, Shanghai, China) with 1 mM phenylmethanesulfonyl fluoride (PMSF, Beyotime) and Phosphatase Inhibitor Cocktail (AbMole Bioscience Inc., Houston, United States). The supernatant was collected by centrifugation at 14,000 r/min at 4°C for 15 min. Cell lysates were subsequently denatured in water with 100°C for 30 min. A standard curve was drawn using the BCA method to determine the protein concentration. Proteins were separated by 10 or 12% SDS-PAGE gel and then were transferred to the polyvinylidene difluoride (PVDF) membrane. The membrane was then blocked in 5% bovine serum albumin (BSA) at room temperature (RT) for 2 h and incubated with a primary antibody at 4°C overnight. Phospho-MEK1/2 (Ser221) (2338), p44/42 MAPK (Erk 1/2) (4695), Phospho-p44/42 MAPK (Erk 1/2) (Th2 202/Tyr 204) (4370), and cleaved-Caspase3 (9661) antibodies were purchased from the Cell Signaling Technology (Danvers, MA, United States). MEK1/2 (11049-1-AP), LC3B (14600-1-AP), P62/SQSTM1 (1840-1-AP), and PTP1B (11334-1-AP) antibodies were purchased from the Proteintech (Wuhan, China). The Tubulin antibody (AT819) was purchased from Beyotime. The PVDF membranes were washed 3 times with TBST buffer and incubated with a secondary antibody horseradish peroxidase-labeled goat anti-mouse IgG (H + L) or goat anti-rabbit IgG (Beyotime) at RT for 2 h. The signals were visualized by using the ECL reagent (Beyotime, China) and the Western imprinting instrument (Clinx Science, Shanghai, China).

### Flow Cytometry Analysis

Cells were treated with drugs for 48 h and then trypsinized and collected for flow cytometry analysis. DMSO treatment was used as negative control. Collected cells were washed with cold PBS twice and incubated in 100 μl binding buffer (BD, San Jose, CA, United States) containing 5 μl 10 μg/mL propidium iodide (PI, Beyotime) and 2.5 μl 50 μg/mL AnnexinV-APC (BD) at RT for 30 min. A Beckman CytoFLEX flow cytometer and FlowJo 7.6 software were used to analyze the apoptosis of melanoma cells.

### mRFP-GFP-LC3 Plasmid Transient Transfection

The mRFP-GFP-LC3 plasmid was purchased from Hanbio Technology (Shanghai, China). The principle of the assay is based upon the different stability of green and red fluorescent proteins. In the lysosome where is under the acidic condition (pH < 5), the fluorescent signal of EGFP could be quenched, however, the mRFP fluorescent signal has no change. In green and red-merged images, autophagosomes are shown as yellow puncta and autolysosomes are shown as red puncta. Increased both of yellow and red puncta in cells indicates that autophagic flux was activated. Increase of yellow without red puncta or decrease of yellow and red puncta indicates autophagic flux was blocked. For transient transfection, the mRFP-GFP-LC3 plasmid were transfected into melanoma cells using ViaFect transfection reagent (Promega, Madison, WI, United States). Transfected cells were grown on coverslips in 24-well plates and treated with TBMS1 alone or combination with other drugs for 48 h. The cells were washed with PBS and fixed with 4% paraformaldehyde for 15 min at RT to make them adhere on the plates. The cells were subsequently permeabilized by 0.1% Triton X-100 for 5 min. About 100 μl 300 nM DAPI (Beyotime) in PBS was used for nuclear staining. Cells were observed under an Olympus confocal laser scanning microscope (Tokyo, Japan).

### Clonogenic Assay

The flat plate colony formation assay was performed as previous report (Dong et al., 2019). Briefly, 1,000 MV3 cells or 1,200 A375 cells per well were cultured in 6-well tissue culture plates and treated with TBMS1 for 10 days. The medium was replaced every other day. After 8 days, cells were fixed with 4% paraformaldehyde and stained with 1% crystal violet for 40 min and slightly washed by PBS for twice. The images of colonies were captured by an Epson scanner. OD 595 nm was obtained by using a microplate reader.

### Tumor Xenografts, Hematoxylin-Eosin (H&E) Staining and Immunocytochemistry (IHC) Assay

One-month-old female nude mice were raised in the specific pathogen free (SPF) room. 1 × 10^6^ MV3 cells suspended in 100 μM saline medium were injected subcutaneously on each side. Four days after injection, the mice were randomly divided into four groups and given appropriate treatment by intraperitoneal injection everyday: control group, TBMS1 group (3 mg/kg/day), CQ group (50 mg/kg/day) and TBMS1 + CQ group. TBMS1 was dissolved in corn oil (Changshouhua, Sanxing Group, Binzhou, China) and CQ was dissolved in saline solution. The length and width of the xenografts were measured by an electronic caliper and the mice body weight were measured by an electronic balance every 2 days. The tumor volume was calculated using the following formula: volume = tumor length × width 2 × π/6. At the end of the experiment, the animals were sacrificed with CO2 at a flow rate of 2 L/min and a replacement rate of 20% volume/min in a 10 liter volume chamber, and then the tumor was excised and weighed. All animal experiments in the current study were preapproved and supervised by the Institutional Animal Care and Use Committees of the Southwest University and the Experimental Animal Care and Use Committees of the Institute of Sericulture and Systems Biology. H&E staining and IHC assay were performed as previous reports (Yang et al., 2019). Anti-Ki-67 (ab15580) and anti-p-ERK (CST4370) antibodies were purchased from the Cell Signaling Technology. IHC positive-signal rate was calculated by the IHC profiler (Varghese et al., 2014) plugged into the Image J software ver.1.46.

### TBMS1 Targets Prediction

The TBMS1 structural information was downloaded from The KNApSAcK metabolomics^1^ and analyzed in the ZINC Database^2^ instructed by previous reports (Irwin and Shoichet, 2005).

### Detection of PTP1B Activity

The method for detection of PTP1B was previously reported (Wang et al., 2012; Przychodzen et al., 2019). In Brief, the MV3 and A375 cells (density at 1 × 10^6^ cells/mL) were treated with DMSO or TBMS1 and incubated for 48 h at 37°C in 6-well plates. The cells were trypsinized and rinsed twice with PBS then suspended at the density of 1 × 10^7^ cells/mL in Cell Lysis Buffer for Western and IP (Beyotime) with 1 mM phenylmethanesulfonyl fluoride (PMSF, Beyotime) and Phosphatase Inhibitor Cocktail (AbMole Bioscience Inc., Houston, United States). The cells were centrifuged at 12,000 *g* at 4°C for 5 min. The supernatants were collected to tubes. The day before the assay, a 96-well microplate were coated with 100 μl of PTP1B capture antibodies at different concentrations (2,4, and 8 μg/mL) and incubated overnight at RT. Then 200 μl Cell lysates were added to the wells then put on a rocking platform at 30 rpm for 3 h. Lysate were aspirated from the wells. The enzyme activity of PTP1B was measured by using chromogenic substrate, para-nitrophenyl phosphate (2 mM). The absorbance was read at 405 nm by the microplate reader. Negative control with para-nitrophenyl phosphate and antibodies but without cell lysates was performed.

### Vector Construction and Transfection

pLKO.1 expressing PTP1B short hairpin RNA (shPTP1B) were constructed by using the double strand of shRNAs below. shPTP1B#1: Forward sequence: 5′-CCGGCTGTGATCGAAGGTGCCAAATCTCGAGATTTGGCA CCTTCGATCACAGTTTTTG-3′; Reverse sequence:5′-AATT CAAAAACTGTGATCGAAGGTGCCAAATCTCGAGATTTGG CACCTTCGATCACAG-3′. shPTP1B#2: Forward sequence: 5′-CCGGCCTAACACATGCGGTCACTTTCTCGAGAAAGTGAC CGCATGTGTTAGGTTTTTG-3′; Reverse sequence:5′-AAT TCAAAAACCTAACACATGCGGTCACTTTCTCGAGAAAGT GACCGCATGTGTTAGG-3′. The negative control pLKO.1-shGFP plasmid was purchased from Addgene. The vectors was transiently transfected in to MV3 and A375 cells using the Lipofectamine^®^ 2,000 Transfection Reagent (11668019, Thermo-Fisher) 24 h before TBMS1 treatment according to the manufacturer’s instructions.

### Real-Time Quantitative PCR Assay

The Real-Time Quantitative PCR (qRT-PCR) was performed as previously reported (Dong et al., 2020). The primers used for detecting PTP1B were designed by previous report (Lu et al., 2012). All primers are shown below: PTP1B-F: 5′-CGGCCACCCAAACGCACATT-3′; PTP1B-R: 5′-GGGGGCT CTGCTTTCCTCTCTG-3′. GAPDH-F: AACGGATTTGGTCG TATTGGG; GAPDH-R: CCTGGAAGATGGTGATGGGAT.

### Statistical Analysis

Graphpad Prism 6.0 were used for statistics analysis. Quantitative data were expressed as the means ± SD. Significant difference was performed by the unpaired, two tailed, student’s *t*-test. A value of *P* < 0.05 was considered statistically significant and was marked with ^∗^ in the figures. *P* < 0.01 was marked with ^∗∗^. *P* < 0.001 was marked with ^∗∗∗^.

## Results

### TBMS1 Inhibits Cell Proliferation in Melanoma Cells *in vitro*

**FIGURE 1:**
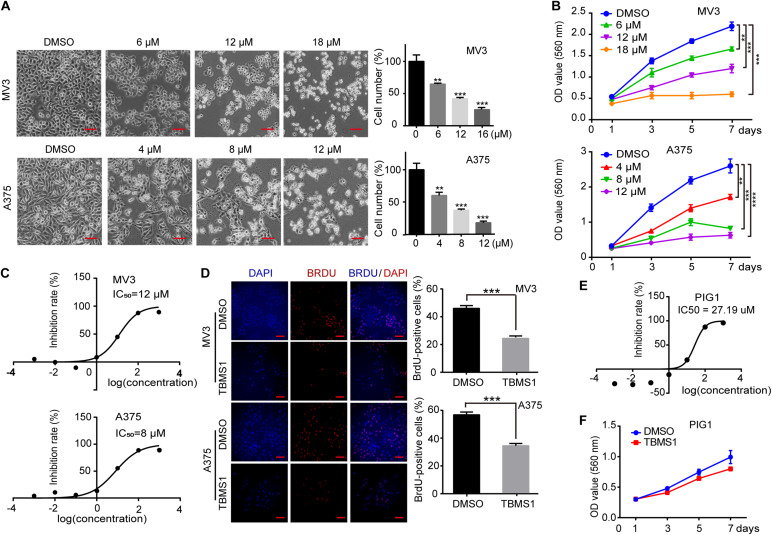
TBMS1 inhibits cell proliferation in melanoma cells *in vitro*. **(A)** Cell morphology of MV3 and A375 melanoma cells after treated with DMSO or TBMS1 in different concentration for 48 h. Cell numbers of MV3 and A375 cells were counted under microscope and then analyzed. Cell numbers of DMSO-treated group were regarded as 100%. **(B)** Viability of MV3 and A375 cells after treated with DMSO or TBMS1. **(C)** IC_50_ of TBMS1 in inhibition of cell proliferation in MV3 and A375 cells. **(D)** BrdU-positive MV3 and A375 cells after treated with DMSO or TBMS1 for 48 h. Quantifications of BrdU-positive MV3 and A375 cells were calculated. **(E)** IC_50_ of TBMS1 in inhibition of cell proliferation in PIG1 cells. **(F)** Viability of PIG1 cells after treated with DMSO or 12 μM TBMS1. A two-tailed unpaired Student’s *t*-test was carried out. *p < 0.05, **p < 0.01, ***p < 0.001, ****p < 0.0001.

To investigate the effects of TBMS1 on melanoma cells, melanoma cell lines (MV3 and A375) were treated with different concentrations of TBMS1 for 48 h. Observed by microscopy, MV3 and A375 cells exposed to TBMS1 showed significant changes in morphology and decrease in cellular number (Figure 1A). MTT assay showed that the viability of melanoma cells treated with TBMS1 was markedly inhibited in a dose-dependent manner, compared with DMSO group (Figure 1B). Based on the results, we calculated the half maximal inhibitory concentration (IC_50_) of TBMS1 in MV3 and A375 cells. Results showed that the IC_50_ of TBMS1 in MV3 and A375 cells were 12 and 8 μM, respectively (Figure 1C). We chose IC_50_ as an indicated concentration for further investigations. BrdU assay showed that DNA synthesis was suppressed in the group treated with TBMS1 for 48 h (Figure 1D). To further confirm the effect of TBMS1 on normal PIG1 cells, we performed IC_50_ assay. The results showed that IC_50_ of TBMS1 on PIG1 cells for 48 h is 27.19 μM (Figure 1E), which is much higher that on MV3 and A375 cells. Besides, 12 μM TBMS1 has no significant effect on cell viability of PIG1 cells (Figure 1F). These lines of evidence revealed that TBMS1 could suppress cellular proliferation of melanoma cells *in vitro*.

### TBMS1 Induces a Partly Disrupted and Cytoprotective Autophagy in Melanoma Cells

**FIGURE 2:**
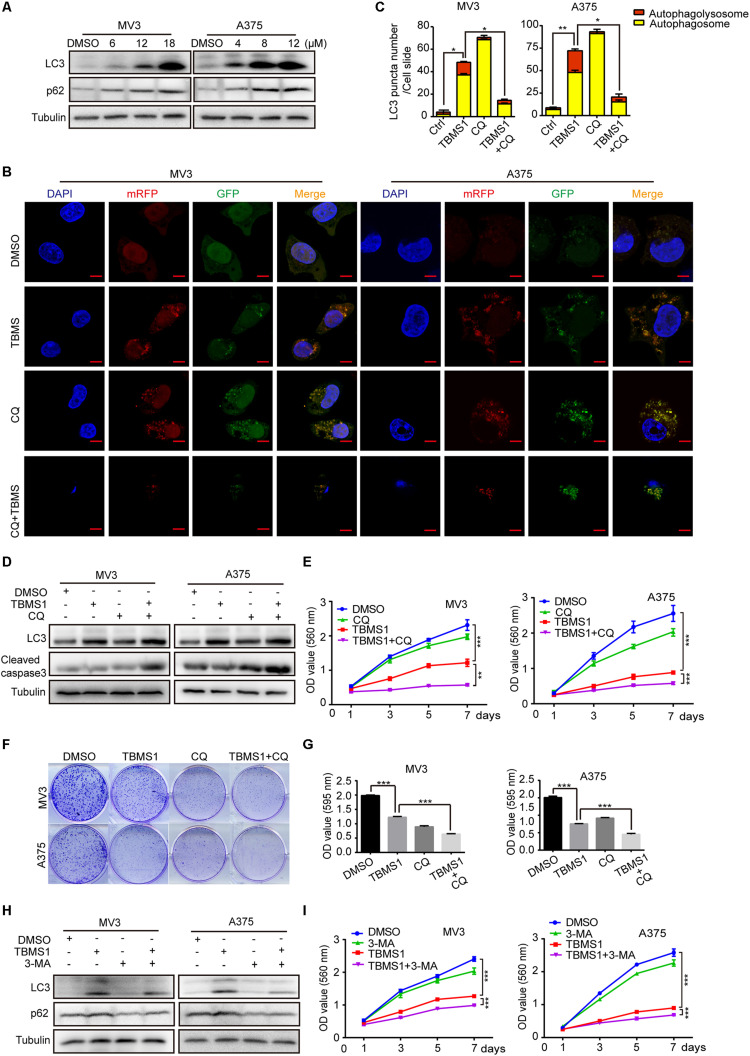
TBMS1 induces a partly disrupted and cytoprotective autophagy in melanoma cells. **(A)** Western blot was performed to detect the expression levels of LC3B-II and p62 in melanoma cells treated with TBMS1 for 48 h. **(B,C)** After transfected with mRFP-GFP-LC3 plasmids, the level of autophagy was tested by immunofluorescence staining assay in MV3 and A375 cells treated with TBMS1 and 20 μM CQ for 48 h. The yellow (autophagosomes) and red signals (autophagolysosome) in every cell per slide were calculated. **(D)** The expression levels of LC3B-II in melanoma cells treated with TBMS1 and 20 μM CQ for 48 h. DMSO was used as control. **(E)** MTT assays were performed to detect cell viability in MV3 and A375 cells under the treatment of DMSO, TBMS1, 20 μM CQ or combination. **(F,G)** Clonogenicity of MV3 and A375 cells treated with TBMS1 and 20 μM CQ. The colonies formed after 10 days’ culture. The quantitative figure of clonogenic assay results. **(H)** The expression levels of LC3B-II in melanoma cells treated with TBMS1 and 2.5 μM 3-MA for 48 h. DMSO was used as control. **(I)** MTT assays were performed to detect cell viability in MV3 and A375 cells under the treatment of DMSO, TBMS1, 2.5 μM 3-MA or combination. A two-tailed unpaired Student’s *t*-test was carried out. *p < 0.05, **p < 0.01, ***p < 0.001, ****p < 0.0001.

As one of the main types of programed cell death in cells, autophagy is essential for cancer cell survival. Therefore, we tried to explore whether autophagy was activated after TBMS1 treatment. LC3B-II is a specific marker of autophagosome formation and accumulation. In the activation of autophagy, LC3B-I is converted to the lapidated LC3B-II form which then merges into the autophagosomal membrane. As a result, LC3B-II transfers from a diffuse pattern to a punctuate pattern. Therefore, the conversion of LC3B is closely related to the status of autophagosomes (Klionsky et al., 2016). SQSTM1/p62, a substrate of autophagy, is delivered to lysosomes to degrade. The rise of p62 can be caused by an increase of protein synthesis or an interrupt of autophagosome turnover (Moscat and Diaz-Meco, 2009). We tested these 2 autophagy-related proteins and it is revealed that LC3B-II and p62 were increased in a dose-dependent manner (Figure 2A), indicating that TBMS1 induced autophagy initiation but the autophagic flux may be interrupted. To further confirm the occurrence of autophagy, we transiently transfected the mRFP-GFP-LC3 plasmids into melanoma cells. The results indicated that LC3B-II positive signals with both yellow and red signals were increased in the experimental groups, revealing that TBMS1 initiated autophagy, but part of the autophagic flux was interrupted (Figures 2B,C).

To further clarify the mechanism of TBMS1-induced autophagy, melanoma cells were treated with TBMS1 in combination with chloroquine (CQ), a lysosomotropic compound that is able to block lysosomal acidification and degradation of autophagosomal components. We pre-treated cells with CQ at concentration of 20 μM for 1 h and then added TBMS1 to treat for another 48 h. The mRFP-GFP-LC3 plasmid assay showed more yellow signals in TBMS1 + CQ group, compared with that of TBMS1 group (Figures 2B,C), indicating that CQ interrupted the autophagy induced by TBMS1. MTT assay represented that the combination led to sharp decline in the cell growth curve (Figure 2E) and the cologenic assay (Figures 2F,G), indicating that the autophagy is beneficial to melanoma cell survival. We also used 3-methyladenine (3-MA), a kind of early autophagy inhibitor, to inhibit the TBMS1-triggered autophagy. Western blot assay results revealed that the protein expression level of LC3B-II and p62 were decreased in the 3-MA group and the combination group (Figure 2H). MTT assay suggested that the suppression effect of TBMS1 was enhanced by 3-MA (Figure 2I). These results showed that autophagy induced by TBMS1 in melanoma cells is partly interrupted, but still remains as a cytoprotective role.

**FIGURE 3:**
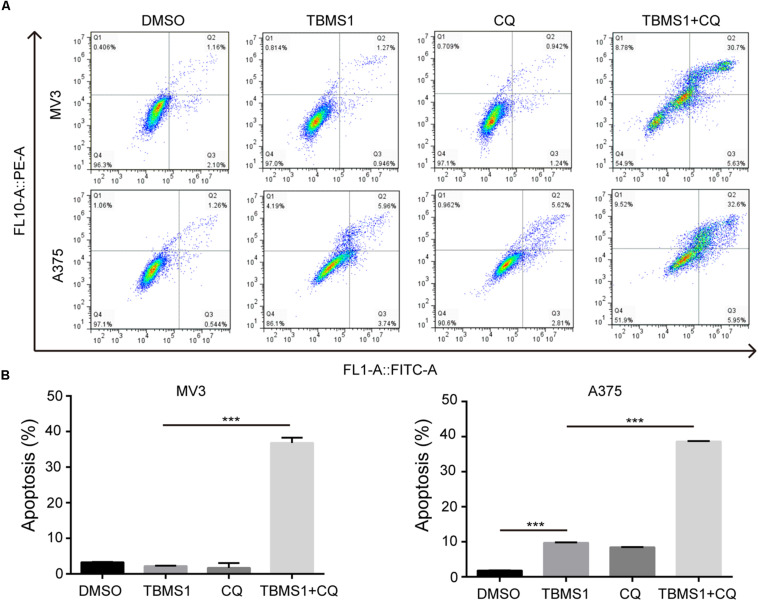
Block of autophagy triggers apoptosis in TBMS1-treated melanoma cells. **(A,B)** Apoptosis was examined in MV3 and A375 cells after treated with TBMS1 and 20 μM CQ for 48 h. DMSO was used as control. Apoptotic rate of MV3 and A375 cells was quantified. A two-tailed unpaired Student’s *t*-test was carried out. *p < 0.05, **p < 0.01, ***p < 0.001, ****p < 0.0001.

In the images of cells treated with TBMS1 and CQ, morphological change like karyopyknosis was also observed. Thus, we checked cells apoptosis by flow cytometry. The results displayed that combination of TBMS1 and CQ triggered apoptosis remarkably, compared with TBMS1 alone (Figures 3A,B). Besides, due to the floating cells observed after the addition of TBMS1 and CQ, we also detected the expression level of cleaved caspase-3, and the result showed that cleaved caspase-3 was elevated remarkably (Figure 2D). All results led to the conclusion that block of TBMS1-induced autophagy enhances the pesticide effect by triggering apoptosis in melanoma cells.

These results confirmed that cytoprotective autophagy is activated by TBMS1 treatment in melanoma cells, and inhibition of autophagy combined with TBMS1 treatment can be a promising combinatory strategy for melanoma treatment.

### TBMS1 Induces Hyperactivation of MEK1/2-ERK1/2 Cascade in Melanoma Cells

**FIGURE 4:**
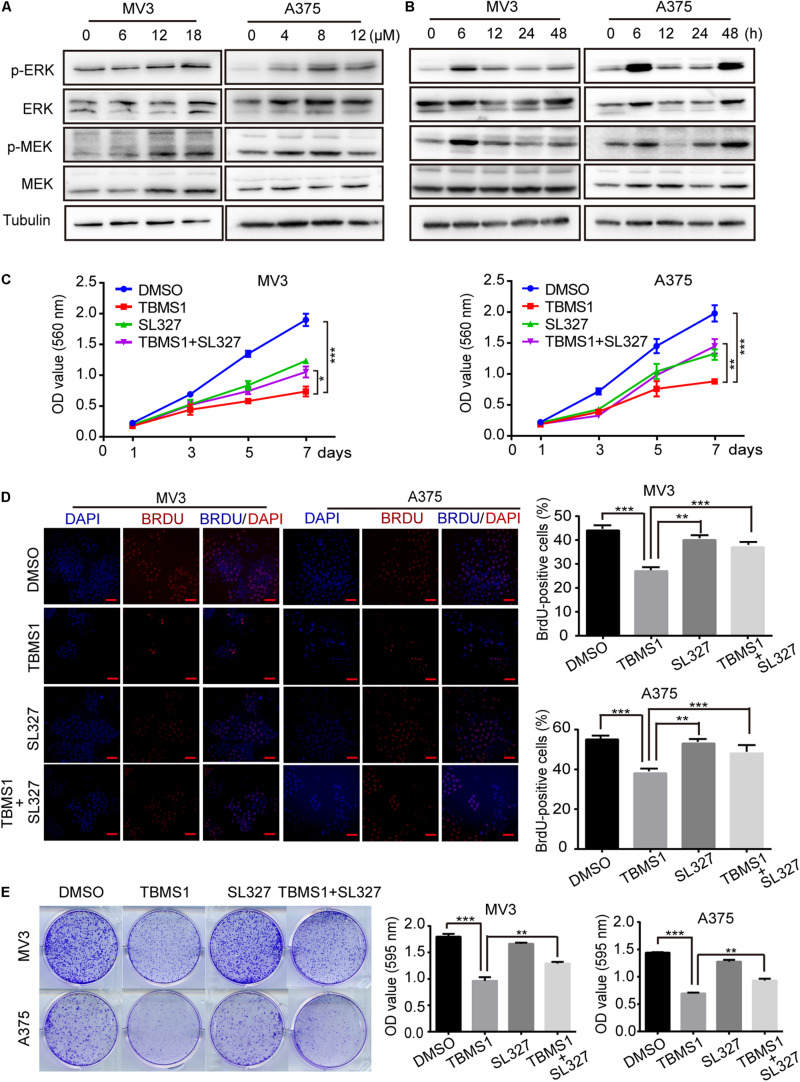
TBMS1 induces hyperactivation of MEK1/2-ERK1/2 cascade in melanoma cells. **(A)** Western blot was performed to detect the expression levels of p-ERK1/2, ERK1/2, p-MEK1/2, and MEK1/2 in melanoma cells treated with TBMS1 for 48 h. **(B)** Western blot results showed the expression level of p-ERK1/2, ERK1/2, p-MEK1/2, and MEK1/2 and in melanoma cells treated with TBMS1 in different time for 0, 6, 12, 24, and 48 h. **(C)** Growth curve of MV3 and A375 cells treated with TBMS1 and 1.25 μM SL327. DMSO was used as control. **(D)** BrdU-positive MV3 and A375 cells after treated with TBMS1 and 1.25 μM SL327 for 48 h. DMSO was used as control. Quantifications of BrdU-positive MV3 and A375 cells were calculated. **(E)** Clonogenicity of MV3 and A375 cells treated with TBMS1 and 1.25 μM SL327. The formation of clones after 10 days culture. On the right is the quantitative figure of clonogenic assay results. A two-tailed unpaired Student’s *t*-test was carried out. *p < 0.05, **p < 0.01, ***p < 0.001, ****p < 0.0001.

High expression level of MEK1/2-ERK1/2 cascade is common in melanoma, because its BRAF(V300E) mutation accounts for 50% of malignant melanoma. Therefore, targeting this pathway as a therapeutic strategy has been widely performed (Savoia et al., 2019). In this study, we also tested the phosphorylated MEK1/2 and ERK1/2 proteins levels (p-MEK1/2 and p-ERK1/2) after treating melanoma cells with TBMS1 for 48 h. However, the results indicated that p-MEK1/2 and p-ERK1/2 were increased instead of reducing in a dose-dependent manner (Figure 4A). This result is opposite to our expectation. To confirm this result, we conducted experiments in cells treated with TBMS1 in a time-dependent manner. As shown in Figure 4B, with time went on, the levels of p-MEK1/2 and p-ERK1/2 increased rapidly at first and then decreased slowly. Nonetheless, the protein levels at 48 h were still higher than at 0 h (Figure 4B). It is reported that hyperactivation of MEK1/2-ERK1/2 cascade is also not benefit for the cell survival (Park, 2014). Therefore, we suspected that TBMS1 can hyperactivate MEK1/2-ERK1/2 cascade. We treated melanoma cells with SL327, a specific inhibitor of MEK1/2, to reveal whether upregulation of MEK1/2-ERK1/2 is involved in effects induced by TBMS1. MTT assay results indicated that 1.25 μM SL327 recovered the growth curve that was inhibited by TBMS1 treatment (Figure 4C). BrdU incorporation assay was performed after cells were treated with SL327 and TBMS1 for 48 h. Results showed that inhibition of MEK1/2 rescued the DNA synthesis reduced by TBMS1 (Figure 4D). Clonogenic assay also illustrated that SL327 recovered the clonogenesis ability of cells which was inhibited by TBMS1 (Figure 4E). In general, MEK1/2-ERK1/2 cascade is induced rapidly by TBMS1 and takes part into the proliferation inhibition induced by TBMS1.

### TBMS1-Induced Autophagy Is Mediated by Hyperactivation of MEK1/2-ERK1/2 Cascade

**FIGURE 5:**
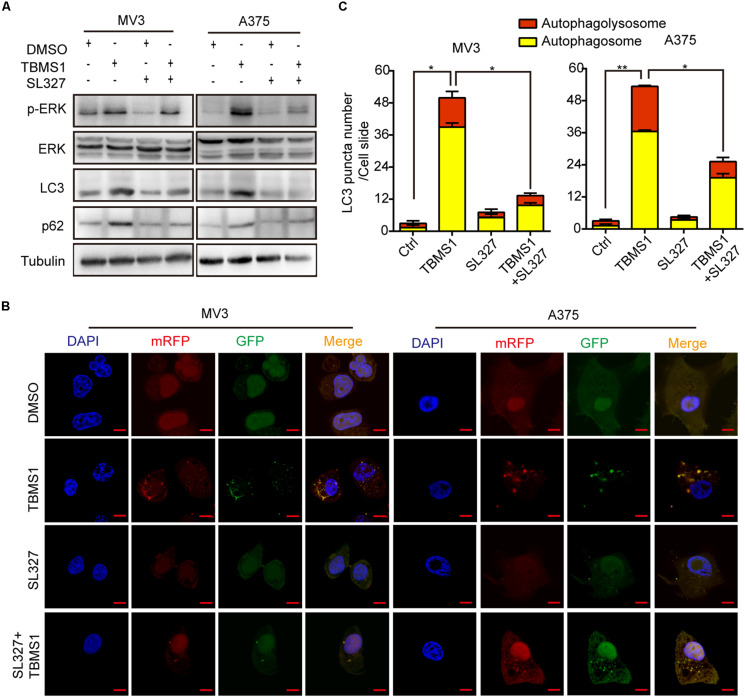
TBMS1-induced autophagy is mediated by hyperactivation of MEK1/2-ERK1/2 cascade. **(A)** Western blot was performed to detect the expression levels of p-ERK1/2, ERK1/2, p-MEK1/2, MEK1/2, LC3B, and p62 in melanoma cells treated with TBMS1 for 48 h. **(B,C)** After transfected with mRFP-GFP-LC3 plasmids, the level of autophagy was tested by immunofluorescence staining assay in MV3 and A375 cells treated with TBMS1 and 1.25 μM SL327 for 48 h. The yellow (autophagosomes) and red signals (autophagolysosome) in every cell per slide were calculated. A two-tailed unpaired Student’s *t*-test was carried out. *p < 0.05, **p < 0.01.

Over activation of MAPK signaling pathway could induce a form of cell death, autophagy (Corcelle et al., 2006; Maiuri et al., 2007). To confirm the results, we used SL327 to inhibit MEK1/2 then examined LC3B-II by Western blot and fluorescence assay. As shown in Figure 5A, after treated for 48 h, SL327 rescued the high level of LC3B-II and p62 induced by TBMS1. The immunofluorescence staining assay images showed that compared with TBMS1 treatment for 48 h, combination of TBMS1 and SL327 induced fewer LC3B-II positive signals with both yellow and red signals (Figures 5B,C). As a result, it was identified that TBMS1-induced autophagy is mediated by hyperactivation of MEK1/2-ERK1/2 cascade in melanoma cells.

### TBMS1 Suppresses Melanoma Cell Tumorigenecity *in vivo*

**FIGURE 6:**
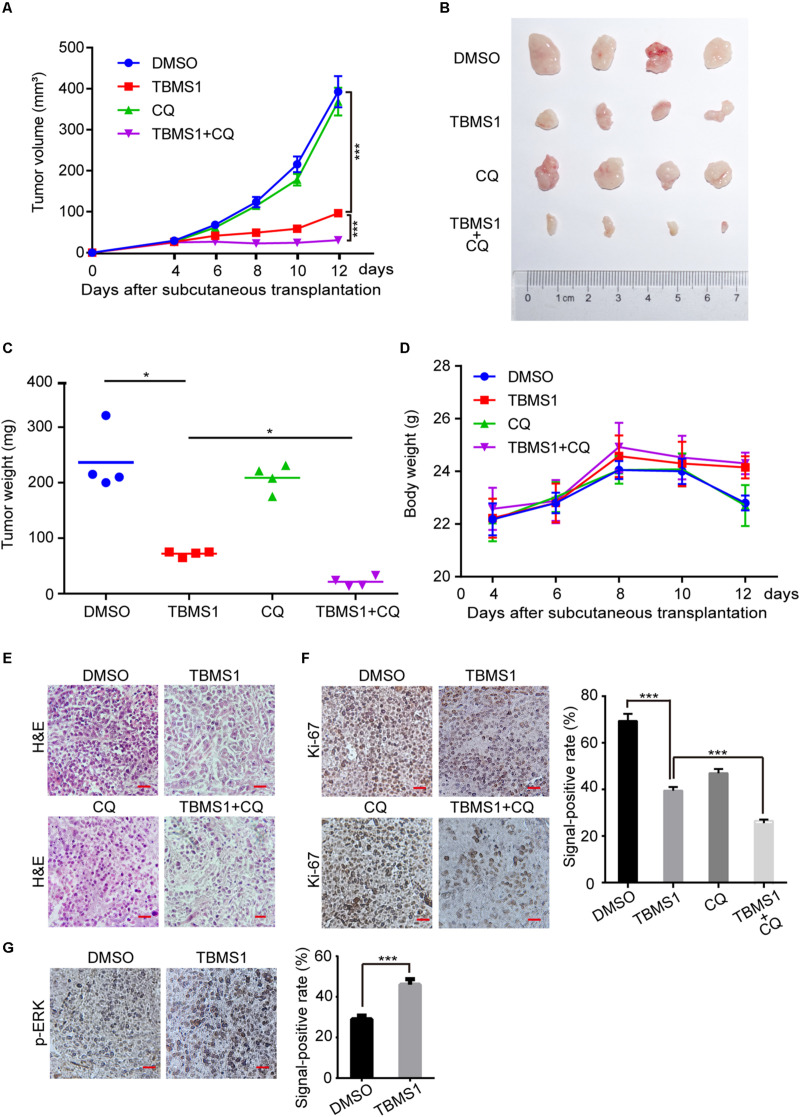
TBMS1 suppresses melanoma cell tumorigenecity *in vivo*. **(A)** The capacity of tumorigenicity was tested in nude mice subcutaneous injected with MV3 cells. After 4 days, mice were treated with TBMS1 (3 mg/kg/day, diluted into saline with 5% DMSO), CQ (50 mg/kg/day, diluted into saline with 5% DMSO), combination with two drugs or control solvent by intraperitoneal. Tumor volume was measured every 2 from 4 days after subcutaneous injection. **(B)** Tumors after resection. **(C)** Tumor weight was detected after the tumor resection. **(D)** The body weight of mice was measured every 2 from 4 days after subcutaneous injection. **(E)** H&E staining of xenografts obtained from subcutaneous injection of MV3 cells under control, TBMS1, CQ or combination treatment. **(F,G)** IHC staining of Ki-67 and p-ERK in xenografts obtained from subcutaneous injection of MV3 cells under control, TBMS1, CQ, or combination treatment. Signal-positive rate was analyzed through IHC profiler in the Image J software. A two-tailed unpaired Student’s *t*-test was carried out. *p < 0.05, **p < 0.01, ***p < 0.001, ****p < 0.0001.

In order to explore the effect of TBMS1 on melanoma cells *in vivo*, we performed subcutaneous tumor assay by MV3 cells in mice. As is shown in Figures 6A,C, tumor volume and weight were decreased in TBMS1 group, compared with DMSO group. What’s more, combination TBMS1 with CQ was able to suppress tumor growth in a more extent, compared with single drug treatment (Figures 6A–C). The body weight of mice presented earlier increase and later decrease trend after TBMS1 treatment. As a result, the average weight of mice in the TBMS1-treated group and combination group was slightly higher than that of the control group, respectively (Figure 6D), indicating that TBMS1 has no obvious toxicity. The H&E assay was conducted to observe the histological changes (Figure 6E). The results showed that the nuclear number in a sing view was less in the TBMS1-treated group and even less in the combination drugs-treated group (Figure 6E). Then, we detected the protein expression level of Ki-67 and p-ERK in xenograft tumors by IHC (Figure 6F,G). From the results, we found that TBMS1 remarkably reduced the expression of Ki-67 and combination with CQ was able to bring down the level further. In addition, TBMS1 increased the expression of p-ERK, which is consistent with the results of our cell experiments. These results concluded that TBMS1 suppresses tumorigenecity of melanoma *in vivo* through hyperactivating MEK1/2-ERK1/2 cascade.

### TBMS1 Plays an Anti-melanoma Role by Targeting PTP1B

**FIGURE 7:**
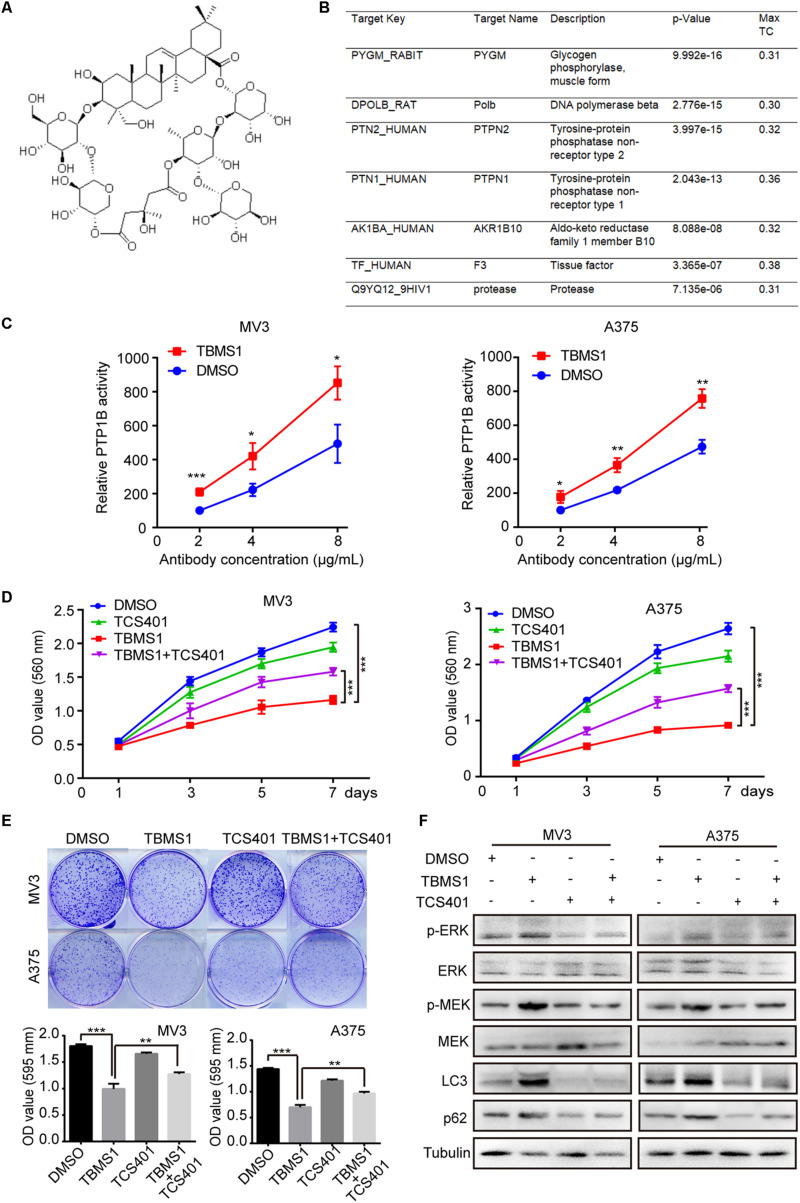
TCS401 rescued TBMS1-induced anti-melanoma activities. **(A)** The structure of TBMS1. **(B)** Target prediction results from ZINC database. **(C)** MV3 and A375 cells were treated with 12 and 8 μM TBMS1 for 48 h and then lysed. Relative activity of PTP1B (% of DMSO + 2 μg/mL antibody) was detected by immunoprecipitation technique and specific substrate. The absorbance was read at 405 nm by the microplate reader. Negative control was sample without PTP1B enzyme. **(D)** Growth curve of MV3 and A375 cells treated with TBMS1 and 2 μM TCS401. DMSO was used as control. **(E)** Clonogenicity of MV3 and A375 cells treated with TBMS1 and 2 μM TCS401. The formation of clones after 10 days culture. On the below is the quantitative figure of clonogenic assay results. **(F)** The expression level of p-ERK1/2, ERK1/2, p-MEK1/2, MEK1/2, LC3B, and p62 in melanoma cells treated with TBMS1 and 2 μM TCS401 for 48 h. A two-tailed unpaired Student’s *t*-test was carried out. *p < 0.05, **p < 0.01, ***p < 0.001, ****p < 0.0001.

The development of bioinformatics has greatly facilitated the prediction of drug targets. A number of structure-based target prediction databases are widely applied. In our study, we used ZINC Database to predict the targets of TBMS1 on the basis of its structure (Figure 7A). The result showed that PYGM, Polb, PTPN2, PTPN1 (also known as PTP1B), AKR1B10, F3, and protease are the likeliest candidates (Figure 7B). Among all the possible targets, it is reported that PTP1B activates ERK1/2 in non-small lung cancer cells and in TAg cells (Dube et al., 2004; Liu et al., 2015). Therefore, we speculated PTP1B is one of the probable targets of TBMS1 and could lead to the induction of MEK1/2-ERK1/2 pathway in melanoma. In order to confirm our speculation, we performed a PTP1B activity assay to determine the impact of PTP1B enzymatic activity after TBMS1 treatment. As illustrated in Figure 7C, TBMS1 significantly activated the enzymatic activity of PTP1B in an anti-PTP1B antibody dose-dependent manner. In addition, TBMS1 has a negative impact on the expression of its downstream MEK1/2-ERK1/2 pathway (Figure 7D). To further confirm the effect of PTP1B, its selectively inhibitor TCS401 was used to suppress the activity of PTP1B. As is shown in Figure 7D and 7E, TCS401 rescued the cell proliferation inhibition and clonogenecity ability suppression led by TBMS1. Besides which, the expression levels of p-MEK1/2, p-ERK1/2, LC3B, and p62 were also recovered in the combination group compared with TBMS1 group (Figure 7E).

**FIGURE 8:**
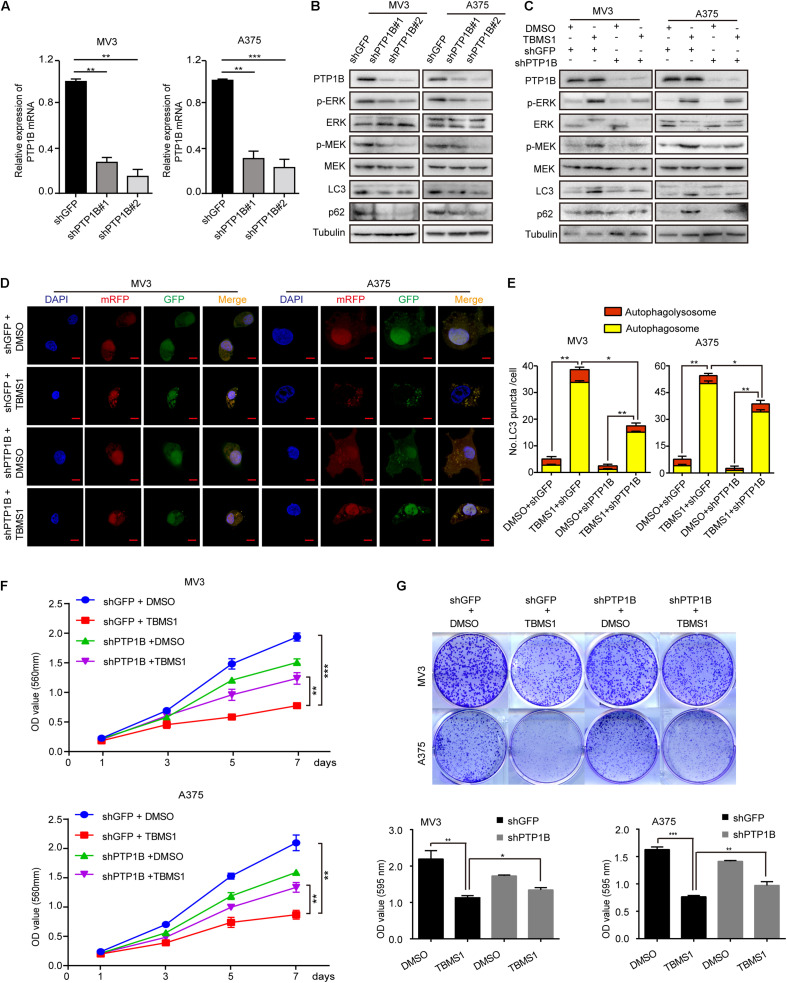
PTP1B knockdown rescued TBMS1-induced anti-melanoma activities. **(A)** qRT-PCR was performed to detect the PTP1B expression in PTP1B-knockdown MV3 and A373 cells. **(B)** Western blot was performed to detect the PTP1B expression in PTP1B-knockdown MV3 and A373 cells. **(C)** Western blot results showed the expression level of p-ERK1/2, ERK1/2, p-MEK1/2, MEK1/2, LC3B, and p62 in PTP1B-knockdown melanoma cells. **(D,E)** After transfected with mRFP-GFP-LC3 plasmids, the level of autophagy was tested by immunofluorescence staining assay in PTP1B-silenced MV3 and A375 cellstreated with TBMS1 for 48 h. PTP1B was transfected in two target cells 24 h before TBMS1 treatment. The yellow (autophagosomes) and red signals (autophagolysosome) in every cell per slide were calculated. **(F)** Viability of PTP1B-knockdown MV3 and A375 cells after treated with TBMS1. DMSO was used as control. **(G)** Clonogenicity of PTP1B-knockdown MV3 and A373 cells treated with TBMS1. The formation of clones after 10 days culture. On the side is the quantitative figure of clonogenic assay results. A two-tailed unpaired Student’s *t*-test was carried out. *p < 0.05, **p < 0.01, ***p < 0.001, ****p < 0.0001.

To further confirm the role of PTP1B in the effect of TBMS1, PTP1B was knocked down by using 2 shRNA sequences which were named as shPTP1B#1 and shPTP1B#2 in both MV3 cells and A375 cells. The shGFP targeting green fluorescent protein (GFP) was used as control. Western blot and real-time qPCR assays confirmed that shPTP1B was effective to inhibit the expression of PTP1B in the two cell lines (Figures 8A,B). It was revealed that PTP1B silencing induced a significant downregulation of MEK1/2-ERK1/2 cascade and p62 expression (Figure 8B). The shPTP1B#2 plasmid was used for subsequent rescuing assays. It is exhibited that TBMS1 led to less increased expression of p-MEK1/2, p-ERK1/2, LC3B, and p62 expression in PTP1B-knockdown groups, compared with that of the TBMS1 group (Figure 8C). What’s more, compared with control cells, the PTP1B-knockdown cells had fewer LC3B-II positive signals with both yellow and red signals after treated with TBMS1 for 48 h, compared with that of the TBMS1 group (Figures 8D,E). The MTT and clonogenic assay also demonstrated that the depression of cell proliferation and clonogenicity ability led by TBMS1 was recovered in PTP1B-knockdown cells (Figures 8F,G).

In brief, our results showed that TBMS1 targets and activates PTP1B, inducing the hyperactivation of MEK1/2-ERK1/2 in melanoma cells.

## Discussion

More than half of melanomas have BRAF mutations (Forbes et al., 2008). Among all types of the mutations, over 80% are a single nucleotide mutation named BRAFV600E (Forbes et al., 2008). BRAFV600E induces activation of the downstream MEK1/2-ERK1/2 (also known as MAPK) cascade (Maurer et al., 2011). In melanoma, the MEK1/2-ERK1/2 pathway is essential for tumor progression (Savoia et al., 2019). However, in some situations, MEK1/2-ERK1/2 plays a dual role in cell survival. ERK signaling can alter cell fates by either excessive inhibition or hyperactivation (Sammons et al., 2019), even in RAS/RAF-mutant melanoma cells (Leung et al., 2019).

Therapy in melanoma has made great progress with the advancement in receptor tyrosine kinase (RTK) inhibitors targeting BRAF/MEK1/2 and immunotherapies (Bommareddy et al., 2017). Although the use of BRAF/MEK inhibitors is essential to BRAF mutations therapy, it lacks efficacy in BRAF WT melanoma (Massa and Kirkwood, 2019). In addition, some patients under the therapy recrudesced with drug-resistance (Sullivan and Flaherty, 2013). About 10–20% of BRAF V 600-mutant melanoma patients have no response to any MEK1/2-ERK1/2 pathway inhibitors (Konieczkowski et al., 2014). In our study, we found that TBMS1 inhibited cell proliferation in both of BRAFV600E mutant A375 cells and BRAF wild MV3 cells by activating MEK1/2-ERK1/2 rather than inhibiting the pathway. Interestingly, ERK activation is involved in lots of cell responses, such as proliferation, differentiation, migration and demise (Murphy and Blenis, 2006). Many studies have suggested a high expression level of ERK in BRAF mutations promotes to increased cell survival (Savoia et al., 2019). On the contrary, an increasing number of studies hold the view that hyperactivation of ERK also facilitates cell demise (Cagnol and Chambard, 2010). For instance, hyperactivation of ERK by multiple mechanisms has also been shown to be toxic to RTK-RAS mutation-driven lung adenocarcinoma cells (Unni et al., 2018). It is reported that high level of MEK1/2-ERK1/2 is able to inhibit cell proliferation as well as low level, in other words, only when the expression of pathway proteins is within a certain range can cell proliferation be promoted (Park, 2014). Importantly, MEK1/2-ERK1/2 hyperactivation has been confirmed to suppress tumorigenesis in B-Raf(V600E) cancers (Atiq et al., 2016).

In our study, we supposed the high level of MEK1/2-ERK1/2 induced by TBMS1 suppressed melanoma cell proliferation. Additionally, the use of MEK1/2 inhibitor could recover the cell proliferation suppression induced by TBMS1. Under the treatment of TBMS1, ERK1/2 phosphorylation increased sharply, then decreased slowly and finally stabilized. We supposed this tendency was due to the negative feedback regulation of ERK. ERK can exert negative feedback at each level of the pathway by phosphorylation (Arat and Harrington, 2014; Hong et al., 2015; Lake et al., 2016; Kidger et al., 2018). The mechanisms of negative feedback regulation play a role in maintaining the pathway stability. Actually, except for ERK, rapid hyperactivation of other oncogenic pathways such as Bcr-Abl also triggers cell death (Dengler et al., 2011). This means that rapidly hyperactivating oncogenic signaling pathways could be a strategy for cancer therapy. In this study, the MEK1/2-ERK1/2 cascade was rapidly hyperactivated within hours by TBMS1 treatment, leading to a toxic stress to cancer cells, indicating that TBMS1 could be a promising anti-cancer drug that is different from traditional strategy.

To elucidate the exact molecular mechanism of TBMS1’s effect in cellular proliferation in melanoma, we performed a bioinformatics analysis based on its 3-D structure. Among all the possible targets, only PTP1B was reported that could activate MEK1/2-ERK1/2 cascade. Mechanically, PTP1B can induce hyperphosphorylation of Src and inhibition of phosphorylation of receptor protein tyrosine kinases (RTKs), thereby promoting RAs-mediated MAPK activation (Dube et al., 2004; Liu et al., 2015). Our further study also confirmed that inhibition or silencing of PTP1B could recover the effect of TBMS1 in the regulation of cellular proliferation and the hyperactivation of MEK1/2-ERK1/2 cascade, indicating that TBMS1 may activate MEK1/2-ERK1/2 cascade through interacting and positively affecting PTP1B activity. Actually, as a classical non-transmembrane protein tyrosine phosphatase, PTP1B can exert both oncogenic and anti-cancerous effects depending on the downstream signaling pathway involved and the cellular context (Lessard et al., 2010). There are many PTP1B inhibitors that are under exploration (Maheshwari et al., 2018). Activation of PTP1B was previously reported to enhance migration, invasion and metastasis murine melanoma B16F10 cells (Martínez-Meza et al., 2019), indicating that PTP1B is also an oncogenic factor for melanoma. We also analyzed the expression of PTP1B in several melanoma cohorts in the Oncomine database and its relationship between patient prognosis, the results also showed that PTP1B is highly expressed in melanoma and high expression of it predicts a worse prognosis (Data not shown). This is consistent with its function in activating MEK1/2-ERK1/2 cascade, because both of them are oncogenic pathways. However, in the present study, MEK1/2-ERK1/2 cascade was rapidly hyperactivated and played an opposite role for cancer cells. It can explain why inhibition of PTP1B by its inhibitor TCS401 or shRNA could also induce cell proliferation inhibition in the present study. However, it needs direct evidence to confirm the direct interaction between TBMS1 and PTP1B.

Since MEK1/2-ERK1/2 cascade is one of the most possible signaling pathways that modulating autophagy, we also found autophagy in the drug-induced cellular response. Coincidentally, ERK activation has a close relationship with autophagy. ERK-dependent autophagic activity is related to specific autophagy markers, such as LC3B-II, beclin1, BNIP-3 and G-interacting protein (GAIP) (Ogier-Denis et al., 2000; An et al., 2006; Cheng et al., 2008; Tong et al., 2015). In addition, accelerating evidence suggests that activation of MEK1/2-ERK1/2 cascade impairs autophagy by disruption of lysosomal function (Corcelle et al., 2006; Kuo et al., 2016). Our study found TBMS1-induced autophagy initiation in melanoma cells by MEK1/2-ERK1/2 signaling pathway. Interestingly, the autophagy induced by TBMS1 was partly interrupted and p62 was increased after TBMS1 treatment. Previous study also showed that TBMS1 induces impaired autophagy in cervical cancer cells via impairing lysosomal enzyme, resulting in p62 accumulation (Feng et al., 2018). The maturation step of autophagy may be inhibited through continuous activation of MEK1/2-ERK1/2 cascade via a mechanism of inhibiting lysosomal activity (Corcelle et al., 2006). However, whether MEK1/2-ERK1/2 cascade associates with TBMS1-induced autophagic flux disruption needs to be further confirmed. Disrupted autophagic flux often showed a cytotoxic effect on cells. Intriguingly, in our study, TBMS1-induced autophagy still remains some cytoprotective functions because not all autophagic flux is impaired. Previous study also showed that TBMS1 induced a cytoprotective autophagy in human breast cancer cells (Jiang et al., 2019). However, they did not show whether the autophagic flux in their study is disrupted or completed.

Autophagy determines cell death and survival. Mechanically, autophagy maintains homeostasis by removing and degrading damaged substrates to recycle and satisfy energy needs (Moreau et al., 2010; Dong and Cui, 2018). Autophagy can promote cell death through excessive self-digestion or by activating other cell death pathways (Kasprowska-Liskiewicz, 2017). Contrarily, under some stimulus, autophagy exerts as a protective process through promoting nutrient and bioenergetic homeostasis, aggregating clearance, p62 regulation, mitophagy, and the removal of intracellular pathogens (Li et al., 2019; Moreau et al., 2010). The cytoprotective autophagy participates in the development of drug resistance and protects cells from chemotherapeutic drugs (Li et al., 2017). Inhibition of this kind of autophagy in cancer cells may result in a bioenergetic shortage (Rubinsztein et al., 2007) or a destruction of cell capacity to remove damaged protein (Kroemer and Jaattela, 2005), which in turn triggers apoptosis (Liu et al., 2017). Therefore, anti-cancer drugs in combination with autophagy inhibitors inhibiting autophagic flux have been recognized to enhance efficacy and sensitize drug-resistant cancer (Li et al., 2017). In this study, we found that TBMS1-induced cell death was potentiated by CQ with a mechanism based on apoptosis, implying the cytoprotective role of TBMS1-induced autophagy.

Since oral administration is not efficient for TBMS1 (Islam et al., 2019), we used intravenous administration for the *in vivo* study. Previous report showed that the acute toxic median lethal dose (LD_50_) of TBMS1 in BALB/c nude mice is 18.7 ± 2.8 mg/kg (Yu et al., 2001). In the *in vivo* study, we used TBMS1 as 3 mg/kg/day, which is much lower than the LD_50_, to confirm its anti-tumor effect. It was shown that TBMS1 significantly inhibited tumorigenesis in the mouse model. Besides, no obvious mice body weight loss was shown. *In vitro* study also showed that the IC_50_ (27.19 μM) of TBMS1 is significantly high in the normal PIG1 cells and treating PIG1 with IC_50_ of TBMS1 in MV3 (12 μM), which is the highest concentration used in cancer cells in this study, showed no obvious toxicity. Previous report also showed that TBMS1 below 40 μg/mL (30.32 μM) has little toxicity in MT-2 human lymphoma cells (Yu et al., 1994).

In summary, it is interesting to note that on the one hand, activation of MEK1/2-ERK1/2 inhibits cell proliferation. However, on the other hand, it also triggers cytoprotective autophagy. The strength of two opposing forces determines the fate of the cells. In this study, TBMS1 interacts with PTP1B, which further hyperactivates MEK1/2-ERK1/2 cascades, leading to cell proliferation inhibition and a partly disrupted autophagy, which remains a cytoprotective role. Importantly, TBMS1 combined with CQ leads to a better result due to the suppression of protective autophagy, which offers a new promising strategy for melanoma treatment.

## Data Availability Statement

The original contributions presented in the study are included in the article, further inquiries can be directed to the corresponding authors.

## Ethics Statement

The animal study was reviewed and approved by the Institutional Animal Care and Use Committees of the Southwest University.

## Author’s Contributions

JD, ZD, YL, and HC designed the study. JD, ZD, LT, MT, KZ, GP, CL, SS, and YZ performed the experiments. JD, ZD, and FZ analyzed the experimental data. JD and ZD wrote the manuscript. YL and HC revised the manuscript. All contributing authors read and approved the final manuscript.

## Conflict of Interest

The authors declare that the research was conducted in the absence of any commercial or financial relationships that could be construed as a potential conflict of interest.

## Footnotes

1 http://www.knapsackfamily.com

2 https://zinc.docking.org/
